# Integrative multicellular biological modeling: a case study of 3D epidermal development using GPU algorithms

**DOI:** 10.1186/1752-0509-4-107

**Published:** 2010-08-09

**Authors:** Scott Christley, Briana Lee, Xing Dai, Qing Nie

**Affiliations:** 1 Department of Mathematics, University of California, Irvine, CA 92697, USA; 2 Department of Biological Chemistry, University of California, Irvine, CA 92697, USA; 3 Center for Mathematical and Computational Biology, University of California, Irvine, CA 92697, USA; 4 Center for Complex Biological Systems, University of California, Irvine, CA 92697, USA

## Abstract

**Background:**

Simulation of sophisticated biological models requires considerable computational power. These models typically integrate together numerous biological phenomena such as spatially-explicit heterogeneous cells, cell-cell interactions, cell-environment interactions and intracellular gene networks. The recent advent of programming for graphical processing units (GPU) opens up the possibility of developing more integrative, detailed and predictive biological models while at the same time decreasing the computational cost to simulate those models.

**Results:**

We construct a 3D model of epidermal development and provide a set of GPU algorithms that executes significantly faster than sequential central processing unit (CPU) code. We provide a parallel implementation of the subcellular element method for individual cells residing in a lattice-free spatial environment. Each cell in our epidermal model includes an internal gene network, which integrates cellular interaction of Notch signaling together with environmental interaction of basement membrane adhesion, to specify cellular state and behaviors such as growth and division. We take a pedagogical approach to describing how modeling methods are efficiently implemented on the GPU including memory layout of data structures and functional decomposition. We discuss various programmatic issues and provide a set of design guidelines for GPU programming that are instructive to avoid common pitfalls as well as to extract performance from the GPU architecture.

**Conclusions:**

We demonstrate that GPU algorithms represent a significant technological advance for the simulation of complex biological models. We further demonstrate with our epidermal model that the integration of multiple complex modeling methods for heterogeneous multicellular biological processes is both feasible and computationally tractable using this new technology. We hope that the provided algorithms and source code will be a starting point for modelers to develop their own GPU implementations, and encourage others to implement their modeling methods on the GPU and to make that code available to the wider community.

## Background

The increasing desire for more integrative and predictive computational models of biological phenomena is offset by the increased computational cost to perform in silico experiments with those models. A simulation that takes many hours or even days to execute tends to inhibit the exploratory nature of modeling just due to the limits of available time. This is exacerbated by the fact that these complex models can also have many additional parameters that must be analyzed to consider their role in the behavior of the model. It is for these reasons that considerable effort is put into technologies, methodologies and theoretical advances to speed up execution without sacrificing model accuracy. Mathematical biological models can be contrasted between continuum models that consider populations of homogeneous biological entities described by differential or integro-differential equations versus discrete models with populations of individual and possibly heterogeneous entities. While continuum models can be more computationally efficient, the need for biological accuracy is encouraging the use of cell-centered and agent-based models with the realization that heterogeneous cell populations more accurately describe multicellular biological processes, e.g. organ development. This heterogeneity is expressed in many ways including cells with spatially explicit shapes that can change over time, cell movement, cell growth and division, cell adhesion, cell-cell interactions, cell-environment interactions and intracellular gene networks coupled to cellular behavior.

Example theoretical and methodological advances include coarse-graining, time-scale separation, and dynamic agent compression. Coarse-graining derives exact or approximate equations for population level dynamics from individual agent interactions, however this approach is often limited to simple forms of interactions [1-3]. Time-scale separation decomposes the model into subcomponents that operate on different time scales thus allowing the slower time scale subcomponents to be simulated less often. This separation might be performed due to prior knowledge about multiple scales in the system, but there are also attempts to determine this separation from the system dynamics [4,5]. Dynamic agent compression aggregates sets of homogeneous agents into a container object which then acts for the agents as a whole [6,7]. Despite these advances, most integrative multicellular biological models still require individual cells and their interactions to be simulated for accurate representation of the biological phenomena.

There is also work to take advantage of new computer technologies. Parallel and distributed computing using programming libraries such as OpenMP [8] and MPI [9] allow for computation to be spread across multiple machines. This architecture is loosely-coupled parallel processing as the machines are connected through a high-speed network where machine-to-machine communication across the network has a high latency associated with it. For example, a parallel implementation of the cellular Potts models uses MPI and a spatial decomposition across multiple machines [10]. Recently, two newer technologies are shifting the performance curve back to tightly-coupled parallel processing where computational units are co-located in hardware with fast communication channels and shared memory. The first technology is multi-core CPUs where manufacturers place more computational units (cores) onto a single processor chip; considerable effort has been put into programming languages, environments and algorithms to allow for a smooth transition from a single sequential processor to multi-core CPUs [11].

The other technology is graphical processing units (GPUs) which are the specialized processors that reside on video display adaptors and drive the graphical user interface of modern operating systems. Because these GPUs do not have to perform many of the generalized tasks that a CPU must perform, they have become highly optimized to perform tightly-coupled data-parallel processing with many, typically hundreds, of independent processor units and specialized memory addressing. GPU algorithms have been developed for many years for computational geometry tasks as part of graphics rendering, but it is only in the past few years where GPUs have been used for other tasks such as sequence analysis [12-14], machine learning [15] and molecular dynamics [16,17]. All of the early implementations had to contend with the constraints and difficulties of the limited programming environment available on the GPU, however this has changed in just the past couple of years. New software toolkits like CUDA and OpenCL have greatly eased the complexity of GPU programming, but there can still be a significant learning curve to achieve peak performance and to scale to large problems.

A recent review describes research to implement a wide spectrum of methods used in systems biology on the GPU, and all of these methods have experienced some level of speedup [18]. Ackermann et al. automatically transform SBML models of biochemical systems into CUDA code to solve the ordinary differentiation equations (ODE) for the system, thus allowing many parameters for those models to be explored in parallel [19]. GPU implementation of the stochastic simulation algorithm can either allow many simulations to be performed in parallel [20] or can parallelize very large models [21], along with the ability to produce many random numbers in parallel [22,23]. Agent-based modeling is one of the more sophisticated methods and has numerous implementation challenges on the GPU to handle dynamic agents and their interactions [24-27]. Of particular interest, the FLAME framework [28] for the GPU [25] allows agent-based models to be declaratively specified using the formal technique of X-machines, and then the corresponding GPU simulation code is automatically generated. Much of this research focuses purely on the implementation of a single method without considering integration of multiple methods. All of the agent-based models implemented on the GPU strictly adhere to the rule-based representation for agent behaviors; though there has been recent work [29] to integrate FLAME with the ODE solver COPASI [30] but it is not implemented on the GPU.

In this article, we will show how to implement using GPU algorithms a number of sophisticated modeling techniques into an integrated biological model that executes orders of magnitude times faster then conventional CPU code. Specifically we provide a model of mammalian epidermal development that incorporates discrete spatially-explicit 3D biological cells that move, change shape, grow and divide. Each cell has an internal gene network that controls behaviors like cell growth and division, and the gene network is coupled to neighboring cell-cell interactions and cell-environment interactions. Furthermore, we provide a set of generic guidelines for GPU programming that when followed will allow modelers to take advantage of GPUs while avoiding many pitfalls associated with the architecture. In the following subsections, we review a number of methods used for modeling biological behaviors and provide some background about the epidermis.

## Modeling Methods for Biological Behaviors

### Cell Shape and Movement

For cell-centered approaches [31,32], three fundamental representations are being used in computational modeling for cell shape: particle, discrete space and continuous space. The particle representation actually does not explicitly represent any cell shape at all but considers each cell to be a point particle. This is the typical assumption made by partial differential equation (PDE) models of large collections of cells where individual cell shapes do not play a role in the behavior of the tissue. Numerical computation of PDEs can achieve significant speedup on the GPU, but they have been discussed elsewhere [33,34] so we will not consider this representation any further. Discrete space or lattice representations divide space into discrete units, then cells occupy one or more spatial units thus defining the spatial extent of the cell. This representation is used by the cellular Potts model [35-37], cellular automata and agent-based models [38-40]. Continuous space, lattice-free or off-lattice representations keep spatial positions as continuous values and then include additional data structures for each cell to describe its spatial extent. There are also agent-based models that use continuous spatial positions [41,42], as well as a few other representations including the center-based method [43,44], the Delaunay-Object-Dynamics method [45], and the subcellular element method [46]. The center-based method represents cells with a center position and a spherical or ellipsoidal shape that may be a rigid body or visco-elastic. The Delaunay-Object-Dynamics method uses a weighted Delaunay triangulation to subdivide space into a set of disjoint Voronoi cells, providing a polygon that defines the cell surface. The subcellular element method uses a collection of discrete particle elements in combination with intracellular spring forces to define a cell.

None of the modeling representations for cell shape completely account for all the complexities of true biological cells, so there are advantages and disadvantages for each depending upon what cell behaviors and interactions are to be modeled, as well as computational tradeoffs. The continuous space methods have a disadvantage over the discrete methods because cell neighborhoods are not defined at discrete lattice points, so interactions involving neighboring cells require a dynamic computation of the neighborhood that is computationally more expensive than an index lookup in a lattice. On the other hand, discrete space methods can have considerable memory requirements as each spatial unit uses up memory space even if a cell does not occupy that space, while the continuous methods only need to minimally store the positions of their discrete elements. However both representations can achieve speedup on a GPU if a significant portion of their calculations can be performed in parallel.

Cell movement and cell shape are generally modeled together. For both discrete and continuous spatial representations, the action of cell movement is similar with either the updating of lattice index values or spatial positions, respectively. Cell movement can be due to internal cell actions, external forces acting on the cell, or some combination of the two. Furthermore, cell movement is realized in different ways depending upon the modeling method being used. For example in the cellular Potts model, cell movement is not explicitly defined but occurs as an indirect result of the process of energy minimization. In contrast, the subcellular element method defines equations of motion, so forces acting on the elements of a cell are directly incorporated into those equations.

In this article, we are going to focus on continuous space methods and specifically the subcellular element method. The subcellular element method is one of the most computationally demanding methods and therefore serves as an excellent benchmark of what can be done with the latest technology. Furthermore, we are going to consider a full 3D spatial environment as this provides the most realistic scenario for studying epidermal development. To our knowledge, this is the first reported attempt to parallelize the subcellular element method.

### Cell Adhesion

Cell adhesion is the process by which adhesion molecules, which are present on a cell's membrane, bind to another cell, the extracellular matrix or some surface. Cell adhesion has many important functions such as maintaining a cell's spatial position, providing structural integrity to a multicellular tissue, creating barriers or tight junctions to prevent movement of fluid between cells, and transmitting environmental signals into the cell. The binding strength of adhesion molecules can vary greatly depending upon the type of molecule and its adhering partner. It is well-known that differential adhesion, whereby two cell types having different adhesive binding strengths between cells of the same type versus cells of different type, can cause cells to physically sort themselves into two distinct populations [47]. When the cellular Potts model was introduced, it was shown to reproduce this behavior [36].

Most modeling methods do not explicitly represent individual adhesion molecules but instead use an aggregate binding strength that is proportional to the cell surface. For example with the cellular Potts model, the number of lattice boundaries shared between two cells is counted and included as a term in the model's energy function. The number of shared lattice boundaries can be used as an absolute count, which can provide larger cells with different adhesive characteristics versus smaller cells, or the number can be normalized by the total cell surface, which can represent a uniform partitioning of some internal cellular resource across the complete cell surface. For continuous space methods, there is no lattice to be counted so a neighborhood calculation needs to be performed. The subcellular element method does this by calculating distances between elements of one cell and another, then incorporating an adhesive force term into the equations of motion for those cellular elements. Similar to the lattice methods, the adhesion strength can be an absolute calculation or normalized by the total cell surface or number of neighbors. A nice feature of the subcellular element method is that elements can have a type associated with them, thus allowing force terms to apply to some element types of the cell but not to others. A typical use of this feature might be to induce polarity within a cell based upon an orientation produced by the adhesion of a subset of elements, or to represent that only a part of the cell's surface area is adhesive.

### Cell Division and Growth

Cell division or mitosis is a complicated biological process involving the duplication of the genome and concluding with the separation of the mother cell into two daughter cells. For many models, the specifics of cell division are not important to the study but the act of division and the resulting growth of the cell population may be very important, especially if the time scale of interest extends across numerous cell divisions. For the subcellular element method, Newman [46] suggests that cell growth can be implemented by adding new elements to the cell over time. If more realistic physical dynamics are desired for the mitotic process, physical constraints can be imposed on some of the elements, for example by defining the spindle axis in the cell and then using it to construct a plane of separation for the two daughter cells. The standard mode for the cellular Potts model is to define a target cell volume as part of the energy function while division is a matter of subdividing the discrete number of spatial units among the two daughter cells.

### Intracellular Gene Network

When employing a cell-centered or agent-based modeling approach, many of the internal details of individual cells can be abstracted away, and representation of a cell's state and associated behaviors is then implemented with state automata [35,48] or axiomatic rules [49,50]. However as molecular biology provides increasing detail about the genes and gene regulations involved in specific cellular behavior, there have been attempts to incorporate these gene networks within individual cells to explicitly drive their behavior and implicitly define their state [51-53]. These models inherently become multiscale as both the spatial and temporal interactions need to be coupled between the cell and intracellular levels. Computational techniques to simulate gene networks include deterministic approaches as represented by a system of coupled ordinary differential equations [54-56] or stochastic approaches that utilize some form of the stochastic simulation algorithm [57-59]. Stochastic algorithms are used when the number of molecules of the biochemical species is small enough such that the stochastic effects play a role in the dynamics of the system; otherwise the deterministic algorithms are preferred as they are computationally more efficient. In this article, we will implement our gene networks using ordinary differential equations (ODEs); however there is research that suggests that stochastic simulations can benefit greatly from GPU implementations as well [20,21].

ODE representation of gene regulatory networks still requires a specific functional form to be chosen for the regulatory interaction. Linear ODEs are commonly used when attempting to infer the network from expression data [60-62]. While linear ODEs are simpler to analyze, they lack the ability to express the more sophisticated behaviors we desire such as cooperativity, thresholds and saturation, so nonlinear ODEs are preferred. For nonlinear ODEs, the two most common representations are Hill-type functions [54,63] and thermodynamic models [64,65]. Thermodynamic models are useful when there is existing knowledge about the promoter structure for the gene allowing binding affinities of transcription factors to DNA as well as combinatorial control to be incorporated. Hill-type functions abstract away the regulatory details and provide a general form for activation or inhibition. We will use Hill-type functions for our gene regulatory networks, however the exact representation used is more of a modeling question and has little effect on the GPU implementation.

## Biological Background

The mammalian epidermis is a tough, resilient protective tissue composed of multiple cell layers that is essential for keeping out harmful microorganisms while also keeping essential fluids inside the organism. Epidermal development proceeds from a single layer of multipotent surface epithelial cells during mid-gestation, to a stratified epidermis consisting of multiple cell types at birth, and finally to a continually self-renewing homeostasis in the mature adult epidermis [66-68]. Both the single layer in the embryonic epidermis and the innermost layer, namely the basal layer, of the stratified epidermis are securely attached to a basement membrane, and are treated as identical populations in this study for simplicity. There are no blood vessels in the epidermis so all nutrients must be transported through diffusion or other mechanisms from the cells in the dermis residing on the other side of the basement membrane. Proliferating cells in the single-layered embryonic epidermis divide symmetrically in the plane parallel to the basement membrane, producing additional cells that attach to the basement membrane, continue to proliferate and increase the overall surface area of the epidermis during embryonic growth [69]. Later in development, basal cells start dividing asymmetrically, in the plane perpendicular to the basement membrane, with one resulting daughter cell maintaining basement membrane contact while the other daughter cell leaves the basement membrane to differentiate and form the suprabasal layers of the epidermis [69]. Finally, the adult stratified epidermis is characterized by a homeostatic process where self-renewing stem cells residing in the basal layer proliferate to replace interior cells while exterior cells are continuously lost to the outside environment.

The epidermis is an advantageous tissue for experimental study due to its accessibility as well as its ubiquity throughout nature. It is also an important tissue for studying how stem cells maintain proliferation and self-renewal over the lifetime of the organism, and for better understanding how errors in those processes can lead to cancer and other diseases. However it is a complicated tissue for computational modeling because it entails a full spectrum of modeling methods to be integrated together into a comprehensive system. The epidermis is a 3D spatial tissue with multiple cell types of differing shapes, behaviors and physical characteristics. There are extensive cell-cell and cell-environment interactions to maintain the structural and functional integrity of the tissue but also to respond to environmental hazards such as wounds and infections. Cells of different types across multiple layers are undergoing various behaviors of growth, division, differentiation and death that must be maintained in proper balance for the health of the epidermis as a whole. Due to the complexity, many models of epidermal development have focused on specific topics such as wound healing [70,71], pathogenesis [72,73], proliferation [74,75], barrier function [76,77] and homeostasis [42,78].

In this article, our goal is not to provide a complete validated model of epidermal development. Instead we use it as a case study for integrative multicellular biological modeling and demonstrate that such models can be efficiently computed using new technological advances such as GPUs. We hope in the future to use our model for the discovery and prediction of underlying mechanisms of epidermal development but for now we focus on the technical aspects of implementing such models in GPU algorithms.

## Results and Discussion

This section is divided into three parts. In the first, we define an integrated model of epidermal development that includes numerous methods, and this model will serve as a case study demonstrating the use of parallel technology for simulation. In the second part, we describe a set of data-parallel algorithms to implement the model of epidermal development on GPUs. We also discuss issues of memory layout and utilization in conjunction with the algorithms. We hope that these algorithms can serve as code templates for modelers implementing their own models, and we further provide source code as Additional file 1 to this article. Lastly, we demonstrate the speedup and scalability that can be achieved using our GPU algorithms, and we show some results from simulations of our epidermis model.

## Model of Epidermal Development

### Cell Shape and Movement

The subcellular element method divides an individual cell into a set of discrete elements or subcellular elements. Biomechanical forces are then defined as interactions for the subcellular elements consisting of intracellular dynamics between elements of the same cell and intercellular dynamics between elements of different cells. The method also includes a weak stochastic component intended to mimic underlying fluctuations in the cytoskeleton of the cell, but we exclude it for this article. The stochastic component is not conceptually difficult to incorporate but it requires consideration of parallel pseudo-random number generation and drawing samples from a multivariate normal distribution. The equation of motion for the position vector $X_{\alpha_{i}}$ of element *i *for cell *α*_*i *_with intracellular and intercellular forces is:

$$\begin{array}{cc} \frac{dX_{\alpha_{i}}}{dt}= & -\nabla_{\alpha_{i}}\sum_{\beta_{i}\neq \alpha_{i}}V_{\text{intra}}\left(\left|X_{\alpha_{i}}-X_{\beta_{i}}\right|\right)\\ & -\nabla_{\alpha_{i}}\sum_{j\neq i}\sum_{\beta_{j}}V_{\text{inter}}\left(\left|X_{\alpha_{i}}-X_{\beta_{j}}\right|\right) \end{array}$$

Part of the modeling process is to determine appropriate potential functions for *V*_*intra *_and *V*_*inter*_. A convenient generalized function is the Morse potential which provides a short-range repulsive force and a longer-range attractive force defined by four parameters (*U*_0_, ξ_1_, *W*_0_, ξ_2_) and the distance *r *between elements:

$$V(r)=U_{0}\exp\left(-r/\xi_{1}\right)-W_{0}\exp\left(-r/\xi_{2}\right)$$

However, biological cells only have a finite range of interactions with other cells, and the Morse potential is typically representing two physical phenomena, spatial exclusivity to prevent cells from overlapping and cell-cell adhesion. The longer-range attractive force is not a biologically realistic interaction across long distances, so we define a positive Morse potential with only a repulsive force that is used to enforce the spatial exclusivity of the cells, which we assume is always active. Cell adhesion is implemented with additional force terms, which allows for greater flexibility in adjusting cell adhesion properties.

$$\begin{array}{c} PV(r)=\{\begin{array}{cc} V(r) & V(r)\geq 0\\ 0 & otherwise \end{array} \end{array}$$

In our subsequent modeling, we will use the standard Morse potential function for the intracellular force, *V*_*intra*_, between elements within a single cell. Based upon the parameters, this will define a typical volume for each cell. We will use the positive Morse potential function for the intercellular force, *V*_*inter*_, between elements of different cells. This will cause cells to repulse each other if they get to close but does not prevent them from drifting apart. Cell adhesion to the basement membrane is incorporated as an additional force term in the equation of motion:

$$\begin{array}{cc} \frac{dX_{\alpha_{i}}}{dt}= & -\nabla_{\alpha_{i}}\sum_{\beta_{i}\neq \alpha_{i}}V_{\text{intra}}\left(\left|X_{\alpha_{i}}-X_{\beta_{i}}\right|\right)\\ & -\nabla_{\alpha_{i}}\sum_{j\neq i}\sum_{\beta_{j}}V_{\text{inter}}\left(\left|X_{\alpha_{i}}-X_{\beta_{j}}\right|\right)\\ & -\nabla_{\alpha_{i}}\sum_{j\in C_{1}}V_{bm}\left(\left|z_{\alpha_{i}}\right|\right) \end{array}$$

The basement membrane is a 2D plane located at Z = 0, and only a subset of a cell's elements are assigned a type, *C*_1_, such that they adhere while the other elements have no adhesive force. As the adhesive force, *V*_*bm*_, acts straight down to the 2D plane, it is only necessary to calculate distance use the *z*-coordinate of an element's spatial position. Table 1 shows the parameter values used in our simulations for the subcellular element method.

**Table 1:** Parameters for Subcellular Element Method in Epidermal Model

Parameter	Value	Description
U_0_	0.3	Intracellular force *V*_*intra *_and basement adhesion *V*_*bm*_

ξ_1_	0.1	Intracellular force *V*_*intra *_and basement adhesion *V*_*bm*_

W_0_	0.12	Intracellular force *V*_*intra *_and basement adhesion *V*_*bm*_

ξ_2_	0.36	Intracellular force *V*_*intra *_and basement adhesion *V*_*bm*_

U_0_	0.3	Intercellular force *V*_*inter*_

ξ_1_	0.05	Intercellular force *V*_*inter*_

W_0_	0.12	Intercellular force *V*_*inter*_

ξ_2_	0.24	Intercellular force *V*_*inter*_

### Intracellular Gene Network

**Figure 1:**
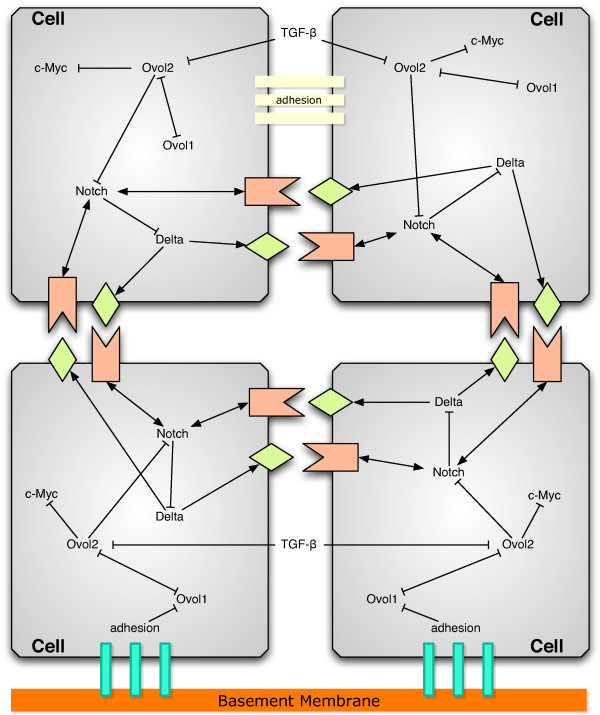
**Diagram of cell-cell and cell-environment interactions coupled with an intracellular gene network within each cell in a model of epidermal development**. Not all interactions have experimental confirmation.

Figure 1 is a depiction of the intracellular gene network, cell-cell interactions and cell-environment interactions for our model. Cell-environment interactions include adhesion to the basement membrane and reception of TGF-β in the extracellular environment, which is known to trigger a signaling cascade that facilitates the growth arrest and subsequent differentiation of proliferating epidermal cells [79,80]. These two environmental interactions act as control inducers for a bistable toggle switch [81] in a dual inhibition circuit between Ovol1 and Ovol2, two transcription factors that are expressed in basal and suprabasal epidermal cells, respectively, and are known to be functionally important in these cells [82-84]. Therefore, cells that adhere to the basement membrane are in the state with low Ovol1 expression and high Ovol2 expression, while cells in the suprabasal layers that no longer adhere to the basement membrane are induced by the environmental TGF-β to be in the state with high Ovol1 expression and low Ovol2 expression. Also included in the gene network is c-Myc, a critical regulator of epidermal cell proliferation and differentiation that is known to be repressed by Ovol2 [84]. Cell-cell interactions include Notch signaling with neighboring cells and cell adhesion. We use a simple lateral inhibition model for Notch signaling that consists of Notch receptors, Delta ligands and bound Notch/Delta complexes [85-87].

The set of equations for the intracellular gene network are:

dNdt=−kaN〈D〉+kdB−dNN+(aBN+bBN1+(cBNB)hBN)(bON1+(cONO2)hON)dDdt=−kaD〈N〉+kd〈B〉−dDD+(aND+bND1+(cNDN)hND)dBdt=kaN〈D〉−kdB−dBBdO2dt=−dOO2+(aVO+bVO1+(cVOO1)hVO)(bGO1+(cGOG)hGO)dO1dt=−dVO1+(bOV1+(cOVO2)hOV)(bAV1+(cAVA)hAV)dMdx=−dMM+(aOM+bOM1+(cOMO2)hOM)A=∑j∈C1|zj|

where the molecular species and interactions being modeled are Notch (N), Delta (D), bound Notch receptor (B), Ovol2 (O2), Ovol1 (O1), c-Myc (M), basement membrane adhesion (A) and TGF-β (G). Parameters include the Hill function production rates for the species, the decay rates for the species, and the association/dissociation rates of the Notch-Delta complex. These parameters and the values used in simulations are described in Table 2. The basement membrane adhesion "molecule" is a conceptual construct representing a cell's adhesion to the basement membrane in order for it to be used as input to the gene network, and simply is the summation of the *z*-coordinate distance for each subcellular element of the appropriate type C_1_. Average Notch receptors and Delta ligands for neighboring cells are represented by ⟨*N*⟩ and ⟨*D*⟩, respectively. For our model, we assume each cell uniformly distributes its Notch receptors and Delta ligands among its neighbors. However it is perfectly reasonable to consider non-uniform distribution, possibly based upon the distances between subcellular elements or specialized types of subcellular elements. Currently we do not implement the diffusible molecule TGF-β in the extracellular space. We assume that the TGF-β concentration is constant in the environment, so all cells see the same value which we define to be G = 0.4.

**Table 2:** Parameters for Intracellular Gene Network of Epidermal Model

Parameter	Value	Description
k_a_	0.0003	Notch (N)/Delta (D) binding rate

k_d_	0.12	Bound complex (B) unbinding rate

d_B_	0.19	Bound complex (B) decay rate

a_BN_	0.01	Bound complex (B) regulation of Notch (N)

b_BN_	1	Bound complex (B) regulation of Notch (N)

c_BN_	1	Bound complex (B) regulation of Notch (N)

h_BN_	-2	Bound complex (B) regulation of Notch (N)

b_ON_	1	Ovol2 (O_2_) regulation of Notch (N)

c_ON_	0.5	Ovol2 (O_2_) regulation of Notch (N)

h_ON_	2	Ovol2 (O_2_) regulation of Notch (N)

d_N_	0.03	Notch (N) decay rate

a_ND_	0.01	Notch (N) regulation of Delta (D)

b_ND_	1	Notch (N) regulation of Delta (D)

c_ND_	10	Notch (N) regulation of Delta (D)

h_ND_	2	Notch (N) regulation of Delta (D)

d_D _	0.006	Delta (D) decay rate

a_VO_	0.1	Ovol1 (O_1_) regulation of Ovol2 (O_2_)

b_VO_	2	Ovol1 (O_1_) regulation of Ovol2 (O_2_)

c_VO_	1	Ovol1 (O_1_) regulation of Ovol2 (O_2_)

h_VO_	2	Ovol1 (O_1_) regulation of Ovol2 (O_2_)

b_GO_	1	TGF-β (G) regulation of Ovol2 (O_2_)

c_GO_	1	TGF-β (G) regulation of Ovol2 (O_2_)

h_GO_	1	TGF-β (G) regulation of Ovol2 (O_2_)

d_O_	1	Ovol2 (O_2_) decay rate

b_OV_	2	Ovol2 (O_2_) regulation of Ovol1 (O_1_)

c_OV_	1	Ovol2 (O_2_) regulation of Ovol1 (O_1_)

h_OV_	2	Ovol2 (O_2_) regulation of Ovol1 (O_1_)

b_AV_	1	Basement adhesion (A) regulation of Ovol1 (O_1_)

c_AV_	1	Basement adhesion (A) regulation of Ovol1 (O_1_)

h_AV_	1	Basement adhesion (A) regulation of Ovol1 (O_1_)

d_V_	1	Ovol1 (O_1_) decay rate

a_OM_	0.1	Ovol2 (O_2_) regulation of c-Myc (M)

b_OM_	1	Ovol2 (O_2_) regulation of c-Myc (M)

c_OM_	1	Ovol2 (O_2_) regulation of c-Myc (M)

h_OM_	1	Ovol2 (O_2_) regulation of c-Myc (M)

d_M_	1	c-Myc (M) decay rate

### Cell Growth and Division

Cell growth and division are handled outside of the GPU in the CPU code. Cells with a low Delta expression, implying that it is in a cellular state with low Ovol1 expression and high Ovol2 expression, indicative of stem cells in the basal layer, undergo growth by adding a new subcellular element every 2000 time steps of the simulation. Adding the new element is a simple matter of increasing the number of elements for that cell then providing an initial spatial position for that new element. We currently put that new element at the cell center from which it can subsequently move to a more appropriate place based upon the forces acting on it.

Once a cell reaches forty subcellular elements then it undergoes division. Cell division is implemented by splitting the forty elements in half, leaving twenty with one daughter cell and twenty in another daughter cell. The spatial positions of the elements are not changed as the subsequent motion will push the two cells apart and adjust their shapes accordingly. One daughter cell is attached to the basement membrane; the other daughter cells is not given subcellular element types for basement membrane adhesion so is free to move away from the basement membrane to become a suprabasal cell and form additional layers of the epidermis. The concentrations of the intracellular gene network products are divided in half between the two daughter cells during division. The non-attaching daughter cell can come under the effect of environmental TGF-β, thus switching the cellular state to high Ovol1 expression and low Ovol2 expression, and enabling Notch signaling to occur between neighboring cells. Dependent upon Notch signaling, some suprabasal cells may still grow and divide.

## GPU Implementation

The primary GPU toolkit currently in use is CUDA [88], which is provided by Nvidia specifically for their graphics cards. Recently, the open standard OpenCL toolkit [89] is provided with Apple's Mac OS X (10.6) Snow Leopard operating system. OpenCL is a more general toolkit for data-parallel programming versus CUDA because it can be used to target both GPUs and multi-core CPUs, and it can be used across a variety of video cards from different vendors. The code we provide will be based on CUDA but it should translate to OpenCL fairly easily as the programming paradigm is very similar.

GPUs are separate devices with their own processors and memory, and do not have direct access to the CPU or CPU's memory. There is a specialized communication pathway for transferring data back and forth between CPU memory and GPU memory. This pathway has a relatively slow bandwidth capability compared to direct access of memory, so it is important to minimize that communication as much as possible when designing GPU algorithms. The typical GPU program has a similar structure to a CPU program and is composed of three main parts as illustrated below:

• Initialization

1. Allocate and initialize model data structures in CPU memory.

2. Initiate connection to GPU device.

3. Allocate GPU memory.

4. Copy data from CPU memory to GPU memory.

• Execution

1. Call GPU kernel functions.

2. Occasionally copy data between CPU and GPU memory.

• Cleanup

1. Free GPU memory.

2. Free CPU memory.

3. Shutdown connection to GPU device.

Because the GPU is a separate device, when the GPU is executing, the CPU is free to perform other computation. More advanced programs can utilize the CPU to concurrently perform tasks while the GPU is running like reading/writing data to disk or pre- and post-processing of data; however for simplicity of presentation in our algorithms we will simply have the CPU wait for the GPU to finish its calculations.

Achieving maximum performance out of GPU algorithms can require some subtle programming tricks and investigating all of these issues is beyond the scope of this article. However we will provide some standard guidelines to follow in the context of the modeling methods that will help obtain significant performance improvements, without needing any sophisticated programming.

### Subcellular Element Method

We will gradually build up the model implementation in parts, adding new features as we go along. The first mechanism to be implemented is the subcellular element method which defines the cells' spatial positions, shapes and movements. Simulating the equations requires picking a numerical scheme to solve the equations over time. Newman [46] used Euler's method but we found we needed to decrease the *δt *in order to maintain numerical stability for some potential functions, therefore we have used the 2^nd^-order Runge-Kutta scheme for all of our algorithms. 2^nd^-order Runge-Kutta requires additional computation and memory because a half time-step intermediate calculation is required, with the previous time-step and the half time-step values used to solve for the next *δt*.

Each subcellular element is independent of the other elements, so a parallel algorithm can calculate the motion equation for each element simultaneously then update the position vectors with their next time step value for all the elements in one step. Therefore we need a data structure to hold the spatial positions for each element and a kernel function that computes the motion equation for a single element. The data structure we will use is a 2D matrix for each spatial coordinate, giving us three matrices for our 3D model, where one dimension of the matrix is the number of cells and the other dimension is the number of subcellular elements.

One limitation of GPU programming is that there is no capability to dynamically allocate memory within the kernel functions executing on the GPU, instead all GPU memory must be allocated beforehand within CPU code and pointers to the GPU memory passed to the kernel functions. This leads to our first guideline for GPU programming.

• **Guideline 1**: Avoid pointer-following data structures such as linked lists and adjacency lists commonly used in CPU code to manage data, e.g. list of cells, which changes in size during the simulation. Use fixed size data structures such as arrays and matrices even if some of the entries would be empty or unused. Such fixed size data structures make it easier to ensure a consistent memory access pattern for kernel functions allowing for greatest throughput.

On the other hand, some algorithms or data are more properly expressed in structures such as trees or graphs. This does not mean these algorithms cannot be used on the GPU, but the traditional pointer-following data structures need to be replaced with array-based representations of those structures along with additional processing such as parallel prefix-sum primitives provided by the CUDPP library [90] to access the data as well as to maintain the data structure.

Given this guideline, we allocate a 2D matrix with the number of columns equal to the maximum number of cells in our simulation and the number of rows equal to the maximum number of elements that any single cell will have. Because cells can have differing number of elements, and there is no simple way to use the coordinate value to indicate a valid element, we also allocate a 1D matrix of size equal to the maximum number of cells that holds the number of elements for each cell. Even though we specify a maximum number for cells and elements, this does not mean that maximum must be fixed. It is possible for the program to re-allocate memory with a new maximum size then copy the old data to the new memory, however it must do this in CPU code and essentially re-initialize the GPU with the new data structures.

Appendix Algorithm 1 shows an initial attempt for a data-parallel implementation of the subcellular element method. It is composed of three functions, **SEM **that allocates memory and executes the kernel function and two kernel functions for the two-step calculation of the 2^nd^-order Runge-Kutta, though we show just one of the kernel functions for brevity as they are very similar to each other. The initial lines of the **SEM **function allocate GPU memory and copy the CPU data to the GPU. We assume that the CPU memory was allocated elsewhere and initialized with initial conditions for the simulation, and the pointers are passed as function parameters. The kernel functions are executed within a loop on the GPU for the two steps of the Runge-Kutta scheme and an update of the position vectors for the next time step. Lastly the GPU data is copied back into CPU memory after the kernel functions have been executed for the desired number of time steps.

The physical processing threads of the GPU can be organized into 1D, 2D or 3D array blocks that are tiled together into a processing grid, which can define a significantly larger number of virtual parallel processors. For example, the Nvidia GTX 285 video card supports 512 physical processing threads but they can tiled together into a grid of over four billion total array blocks thus providing one trillion virtual parallel processors. Typically the virtual processors are organized according to the data structure being operated upon because a unique identifier number is assigned to each thread that can be used to determine a unique index number into the data structure. Our spatial coordinates are organized in 2D matrices, so we define a 2D block of 16 × 16 threads tiled together into a grid with dimensions based upon the maximum number of cells and elements. Note that this construction of blocks tiled into grids is specific to the CUDA toolkit. OpenCL is more general in that it just requires the number of desired virtual processors, specified as a 1D, 2D or 3D array, and OpenCL maps them appropriately to the underlying available hardware, which can vary in the number of physical threads available and the maximum number of virtual processors.

The first two lines of the kernel function in Appendix Algorithm 1 (**sem_kernel_F1**) shows how the identifier number for the processor thread is translated into a cell number and an element number that will be used as index values for the 2D matrices. The processing threads are organized according to the maximum number of cells and elements, so the next two lines of the kernel function checks that the numbers are within the actual number of cells and elements for that cell, and if not then the kernel function returns immediately. It is important to have bound checks such as these in all kernel functions, both to prevent useless computation but more critically to prevent out-of-bounds memory writes. The configuration bounds of the processing threads may not align exactly with the data structure bounds, and there is no memory bound protections on GPUs, so such out-of-bounds memory writes can easily overwrite other data. The remaining code iterates through the elements of the same cell and calculates the intracellular force potentials, then it iterates through the elements of other cells and calculates the intercellular force potentials. Finally, it calculates the adhesion force and combines them together to update the spatial position of the element.

Appendix Algorithm 1 corresponds to the naïve *O(N^2^M^2^) *algorithm where *N *is the number of cells and *M *is the number of elements. This algorithm actually performs well and provides a significant improvement over Newman's time (see the Testing section for complete timing results), but the algorithm does not provide the level of scalability that we desire. As the number of cells increase, the quadratic nature of the algorithms starts to dominate, much of the computation is wasted because most elements are too far apart from each other to exert any significant force; it is really only the nearby elements that matter. Sophisticated methods and data structures have been utilized in CPU code to identify nearby elements. Newman uses a sector technique that maintains a look-up table of the list of elements located within a discrete partitioning of space, elements then just need to perform calculations with elements in the neighboring sectors.

One may think that implementing such sophisticated data structures in the GPU is necessary to achieve greater speedups, but in fact that ends up being counter-productive. We implemented Newman's sector method on the GPU and found it to actually go slower than CPU code (data not shown). For one, it tends to violate Guideline 1 that says to use simple fixed-sized data structures, but it also greatly complicates the kernel function, which leads to our second guideline.

• **Guideline 2**: Simpler kernels execute faster. Break apart complicated kernel functions into simpler kernels using memory to hold intermediate values. Avoid nested loops, especially when the loop bounds are variable. Compilers can do a better job of optimizing simpler kernels, and simple loops can be unrolled and/or instructions re-ordered to maximize coalesced memory access.

On the other hand, too many overly simple kernels can actually perform worse than a single complicated kernel because of the additional overhead imposed by launching more kernels, so the opposite extreme should be avoided as well. It is a good idea to modularize your code into functions when it is feasible; the accompanying source code has examples for how some operations like distance and boundary condition calculations are put into functions. Functions on the GPU are inlined into kernels during compilation, so they do not incur the stack and execution time as in CPU code. The resultant benefit is kernels can be constructed by combining function calls together, and kernels can be more easily adjusted by adding or removing functions.

Appendix Algorithm 2 is the result when we split up the intercellular potential calculation into two simpler calculations. First we calculate a center point for a cell from the positions of all the cell's elements. Second when calculating the intercellular potential, we use a cell's center point to determine if the cell is too far away (as defined by some cutoff value) then skip that cell completely. While in the worst-case scenario this can be as expensive as Appendix Algorithm 1, typically a cell only has a few nearby neighbors making its complexity *O(N^2 ^+ N^2^M)*. With *N *parallel threads for the cell center calculation and *NM *parallel threads for **SEM**, this new algorithm is essentially linear with *O(N) *complexity. The Testing section shows the algorithm performs very well with simulations of 5000 cells each with 20 elements executing on the order of minutes, instead of hours as Newman predicts with his CPU code [46].

### Boundary Conditions

Biological modeling papers typically mention the boundary conditions they assume while describing the model, but less frequently is there discussion about the implications those boundary conditions have on the computational implementation of the model. Boundary conditions can be either periodic, no-flux, or no-boundary. Periodic boundary conditions are used when the model represents just a portion of a larger tissue, and mirroring of the model allows objects near the boundary to perceive that the system continues beyond the boundary and thus is larger then it really is. No-flux boundary conditions act as a barrier that prevents the passage of any objects past the boundary, and this might reflect a physically imposed boundary such as the edge of a petri dish or a biological boundary such as the edge of the tissue. Sometimes no-flux boundaries might be considered leaky in that they allow one-way passage of objects across the boundary, essentially acting as a sink or source. No-boundary conditions imply that there is no boundary allowing the domain to increase in size, or there may truly be a boundary but the objects in the model never reach it so it seems non-existent.

There is an interesting dichotomy in that periodic boundary conditions have a simple implementation for lattice models while no-boundary conditions have a more complicated implementation. In contrast, lattice-free models have a simple implementation for no-boundary conditions and a more complicated implementation for periodic boundary conditions. For no-flux boundary conditions, there is a simple implementation for both lattice and lattice-free models. The contrast between the two models is due to the spatial representation. For lattice models, the spatial domain is defined by the extent of the underlying lattice that typically has an underlying memory data structure of the same size, so expanding the spatial domain as with no-boundary conditions requires that memory data structure to be increased, which is an expensive operation. On the other hand, a periodic boundary requires just a simple calculation of an index value based upon the current size of the lattice. For lattice-free models, there is no explicit representation of the spatial domain encoded in the position vectors of objects, so they are free to change values without constraint under no-boundary conditions. However, periodic boundary conditions requires a mirror of the model to be presented on the other side of boundary with those mirror objects being included in all spatially-based calculations.

The algorithms we presented for the subcellular element method in the previous section are for no-boundary conditions, however we want periodic boundary conditions for our model of epidermal development because it is just a small part of the larger epidermis. Specifically, only the two horizontal planes have periodic boundaries while the vertical plane has a no-flux condition for the bottom with the basement membrane and no-boundary for the top. This means we require eight mirrors of the model, the four sides and the four corners, so we have eight additional calculations required for each subcellular element. We do not show the algorithm for implementing periodic boundary conditions, as the tedious calculations are lengthy but straightforward, but the accompanying source code can be consulted for details. In the Testing section we provide timing information for both no-boundary and periodic boundary conditions. Periodic boundary conditions are slower as expected, but not nearly as slow as the theoretical 9 × based on the number of additional calculations, in large part because many unnecessary calculations are avoided due to the distance cutoff.

### Intracellular Gene Network

Because each cell has its own intracellular gene network with the corresponding set of concentration values for the various molecular species and interactions, the ODE calculations for each cell can be performed in parallel. However in our epidermal model, cells are coupled with their neighbors through Notch signaling so the calculations across cells need to be synchronized in time for correctness. We reuse the cell center calculation from Appendix Algorithm 2 for determining a cell's neighbors, and according to Guideline 2 we use a separate kernel function to accumulate neighbor values for each cell and save them in memory. We allocate a set of 1D arrays of size equal to the maximum number of cells to hold the accumulated neighbor values. Furthermore, we allocate a 2D array with column size equal to the maximum number of cells and row size equal to the number of species (seven for our model) to hold the concentration values for the ODEs. Because the parameters are the same for all cells and do not change, a 1D constant array is allocated for them. Access to read-only constant memory is nearly as fast as register access, so it is much better for holding parameters values than global read/write GPU memory, but it is limited in size. For example, the GTX 285 has 64 k bytes of constant memory capable of holding 16 k floating point values, which is more than sufficient for parameters shared among all cells. However if heterogeneous cells are desired, each with a different set of parameter values, then the constant memory may not be big enough for large simulations.

Appendix Algorithm 3 shows how the ODEs are calculated for each cell, again using the 2^nd^-order Runge-Kutta numerical scheme, thus giving us four functions. The **SEM **function, as before, allocates GPU memory, copies CPU data to the GPU then calls the kernel functions for the ODEs. One kernel function accumulates the neighbor values, while the other two kernel functions perform the two steps of the 2^nd^-order Runge-Kutta though we show just one of these functions for space considerations.

While Appendix Algorithm 3 works well for the small intracellular gene network in our epidermal model, it will not scale up to ODEs containing hundreds or thousands of equations. The reason for this is not immediately obvious. Looking at Appendix Algorithm 3, it seems perfectly reasonable to keep adding a few additional lines of code for each ODE equation; even though the kernel function may get long, the calculations are simple and straightforward. Eventually though, the GPU will run out of registers.

The underlying reason is that the compilers for GPU code, **nvcc **in CUDA for example, optimizes code for doing memory writes and aggressively uses registers to hold intermediate values. Specifically memory writes such as saving the final calculation for an ODE equation might be deferred until later so that writes can be performed which best utilizes the memory bandwidth. This is all legal so long as this reordering of instructions doesn't change the program semantics, and such techniques are used heavily by CPUs to keep their pipelines full and their cache hit rates high. The difficulty is that there is no stack on GPUs, so these pending memory write values are stored in registers, adding more ODE equations increases register usage until eventually register overflow occurs. Overflowed registers trigger fail-safe operation on the GPU by putting the data into global GPU memory, which greatly disrupts the intended optimizations and causes the GPU code to run extremely slow. Furthermore, the compiler cannot account for the register overflow situation during compilation of the source code because it does not know how many processing threads will be running concurrently. If register overflow occurs, one can decrease the number of concurrent processing threads, but this reduces parallelism which eventually defeats the speedup advantage of the GPU. This leads to our next guideline.

• **Guideline 3**: Local variables and registers are a limited and precious resource shared among all concurrent threads. Organize your code to use few local variables and reuse those variables when possible. Achieve greatest GPU occupancy by maximizing the number of concurrent threads while minimizing the register usage of each thread without exceeding the total available registers.

This guideline is an extension of Guideline 2 which suggests simpler kernels, as a more complex kernel will tend to use more registers. For the calculation of a large set of ODEs, the single kernel function that calculates all equations would be split into multiple kernel functions where each calculates only a small number of ODE equations. Unfortunately achieving optimal GPU performance is not such a straightforward affair as just writing simple kernels. A better performance metric is to consider GPU occupancy. GPU occupancy is defined by the combination of the number of threads, the number of registers used by each thread and the amount of shared memory used by each thread. Maximizing occupancy entails writing kernel code and executing the kernel in a configuration that maximizes the number of threads while minimizing the number of registers and amount of shared memory used by each thread. CUDA comes with a profiler application that can provide various performance statistics helpful in optimizing GPU code, and we present some results from the CUDA profiler for Appendix Algorithm 3 in the Testing section.

**Figure 2:**
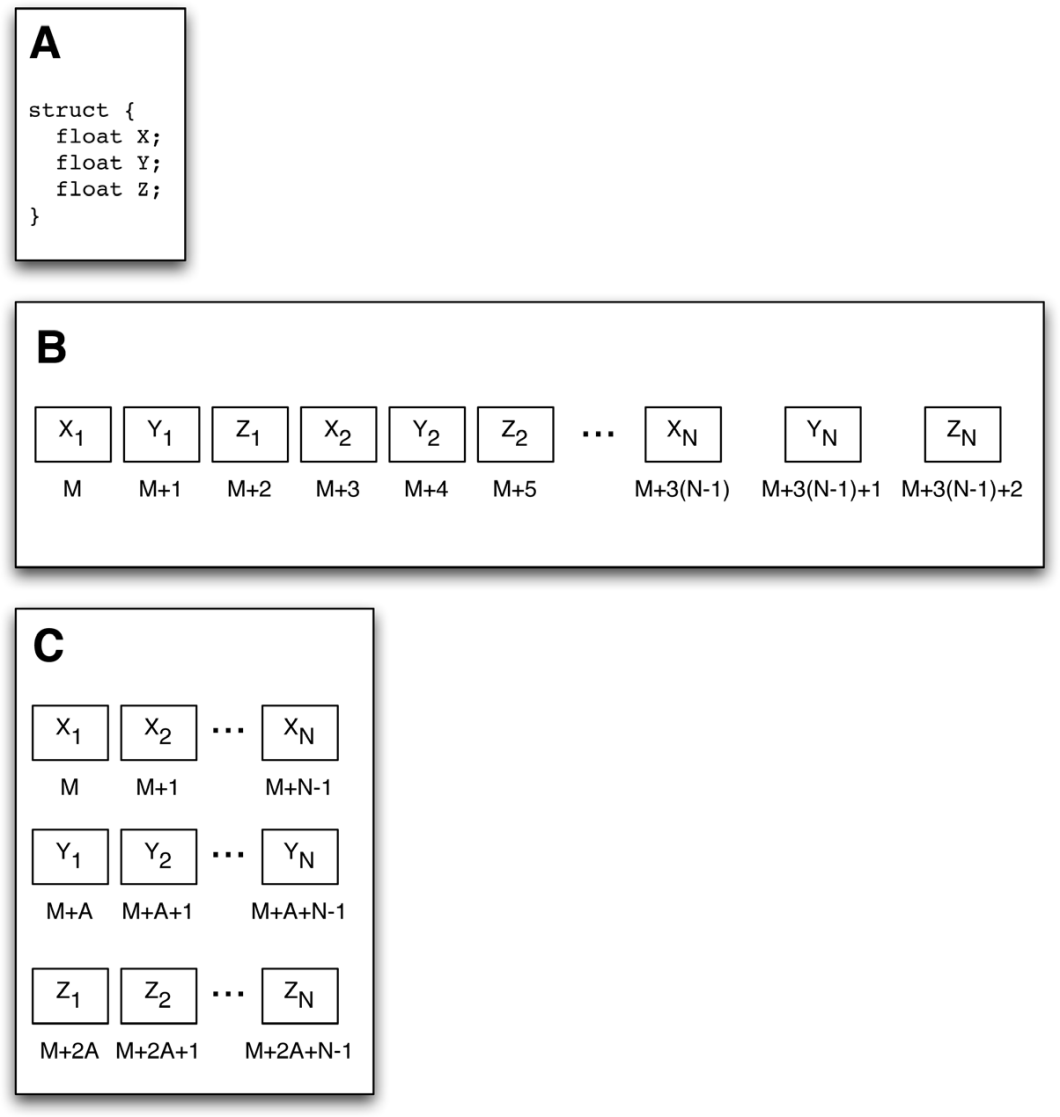
**Data structure memory layout**. (A) An example data structure containing three variables to hold 3D spatial coordinates. (B) Memory layout of the variables when N data structures are allocated together in a CPU program. The variables for each data structure are grouped together in sequential memory locations. (C) Preferable memory layout of N data structures for a GPU program. Each variable in the data structure are grouped together in sequential memory locations, and each variable group is padded to value *A *to insure alignment requirements. Alignment can be handled automatically, such as with CUDA's cudaMallocPitch function, and the alignment value must be used when calculating array indexes.

Modern programming languages have encouraged the design where data is encapsulated together into classes or structures. This design has a direct impact on how data is organized in memory as illustrated in Figure 2. This memory organization while acceptable for CPU programs will lead to poor performance for GPU programs. In the algorithms presented, we have followed a strict guideline for our data structures:

• **Guideline 4**: Organize data structures in global memory such that concurrent threads access contiguous sequential memory locations. This access pattern to global memory will help maximize coalescence. Consider using the other memory types available such as shared, texture or constant memory, as they can be significantly faster than global memory.

Memory coalescence is when simultaneous accesses to global memory by threads can be combined together into fewer memory transactions. By maximizing memory coalescence, global memory bandwidth is used most efficiently. However GPUs have multiple types of memory, while in our code we only use constant and global memory. These other memory types such as shared, texture and constant memory are limited in size and functionality, but they are faster than global memory for read operations and thus can provide additional speed improvements. Use of different GPU memory types may require optimizing for different access patterns, e.g. shared memory needs to minimize bank conflicts.

The 2D array in Appendix Algorithm 3, which holds the concentration values for the ODEs, is specifically designed so that the columns are the cells while the rows are the different species. This is because each thread performs the ODE calculations for each cell in parallel, so the concurrent kernel functions will access sequential memory locations. If the 2D array was transposed such that the columns are the different species while the rows are the cells, then concurrent threads no longer access sequential memory locations and memory coalescence is compromised. We have performed experiments (data not shown) that indicate a 2-3 × speed difference can be incurred just due to this array orientation.

## Testing

We conducted timing experiments for three progressive model implementations starting with the subcellular element method, then adding the intracellular gene network and finally including cell growth and division. For our discussion, these models are named the movement model, the gene network model and the full model. Table 3 shows the execution time for each model for a different starting cell population. Simulations were run on a dual processor 2.8 Ghz quad-core Intel Mac Pro with an Nvidia GTX 285 graphics card; the GPU did not have a monitor attached so it could be completely dedicated to computation. Execution times are reported for both periodic and no-boundary conditions as well as using the naïve and the center point implementations for the subcellular element method corresponding to Appendix Algorithm 1 and 2 respectively. The execution times are the average of three simulations except for the full model where just a single simulation was run. For the movement and gene network models, the timing is for 3000 iterations for comparison against Newman's CPU algorithm, and each cell has 20 subcellular elements. For the full model, 3000 iterations is not sufficient time for the cells to grow and divide, so we report 130000 iterations which allows for multiple cell division cycles and increases the cell population size approximately five fold. The simulations of the full model correspond to the epidermis growing from a single layer to multiple layers.

**Table 3:** Execution Time of Algorithms by Cell Population Sizes

Method	128 cells	250 cells	500 cells	1000 cells	5000 cells
Movement					

Newman	180s				

No boundary	16s	33s	80s	218s	Kernel limit

Periodic boundary	43s	85s	263s	793s	Kernel limit

Center, No boundary	10s	20s	33s	50s	550s

Center, Periodic boundary	26s	48s	79s	125s	944s

					

Movement, Gene Network					

No boundary	18s	38s	86s	241s	Kernel limit

Periodic boundary	46s	87s	286s	811s	Kernel limit

Center, No boundary	13s	24s	42s	72s	630s

Center, Periodic boundary	29s	51s	88s	146s	1038s

					

Full Model					

Center, No boundary	18m19s	41m59s	93m38s	239m34s	

Final cell count	623	1159	2377	4735	

Center, Periodic boundary	61m14s	151m25s	286m15s	573m55s	

Final cell count	582	1059	2286	4585	

For the movement model using the center point implementation, Table 3 shows that our GPU algorithm for 128 cells is 18 × faster than Newman's CPU algorithm which he ran on a 2 Ghz PC [46]. The speedup could be considered even greater as Newman used a single step Euler scheme while we used a two-step Runge-Kutta scheme. As the number of cells is increased, especially starting at 500 cells, the naïve implementation starts to perform poorly until it exceeds the kernel runtime limit at 5000 cells while the center point implementation scales well allowing 5000 cells to be simulated in under 10 minutes for no-boundary conditions. Inclusion of the intracellular gene network adds a small amount to the total execution time indicating that the subcellular element method dominates the computation.

The execution time for the full model takes longer as we run for over 40 × more iterations, but the results indicate even scaling across the initial cell population sizes as well as the increase of the cell population during the simulation. We did not run simulations for the initial cell population size of 5000 as this would entail final cell counts of almost 30000. While simulations for large cell populations do take a long time to run, they actually become feasible using the GPU algorithms; sequential CPU code could take numerous days to run the same simulation. Even more significant is that the simulations for smaller initial cell populations, values that are reasonable for studying epidermal development, can be executed in a couple hours or less. Periodic boundary conditions add a significant amount of time to these longer running simulations, so additional optimization is worth investigating.

We used the CUDA profiler tool to gather statistics for the kernel functions for the gene network model with the center point implementation, periodic boundary conditions, and 128 cells. Table 4 shows the profiler results of percentage of GPU time, number of registers, occupancy, and percentage of divergent branches for each kernel function. The kernels for the subcellular element method clearly are dominant using almost 90% of the GPU time. Occupancy analysis indicates that the number of registers limits the subcellular element method kernels while the other kernels are limited by the block size. The occupancy for those other kernels is low, so performance can be improved by increasing the number of threads per block. The subcellular element kernels could be improved if the number of registers could be reduced, thus allowing more threads to be concurrently executed. A divergent branch occurs when the threads take different code paths out of a branching instruction, e.g. if-then-else, thus forcing a sequential instruction flow and reducing concurrency. The percentage of branches that diverge is under 1% for all the kernels indicating that the algorithms maintain good concurrency. Overall the profiler results suggest that implementing additional optimizations might improve performance even further.

**Table 4:** CUDA Profiler Results for Kernels in Movement/Gene Network Model

Kernel	%GPU time	Registers	Occupancy	%Divergent Branches
skin_moveKernel_F1	44.85%	35	0.25	0.85%

skin_moveKernel_F2	44.83%	32	0.5	0.85%

skin_neighbor_kernel	8.76%	20	0.125	0%

skin_center_kernel	0.42%	19	0.125	0.56%

skin_kernel_F1	0.34%	28	0.125	0.64%

skin_kernel_F2	0.34%	28	0.125	0.64%

**Figure 3:**
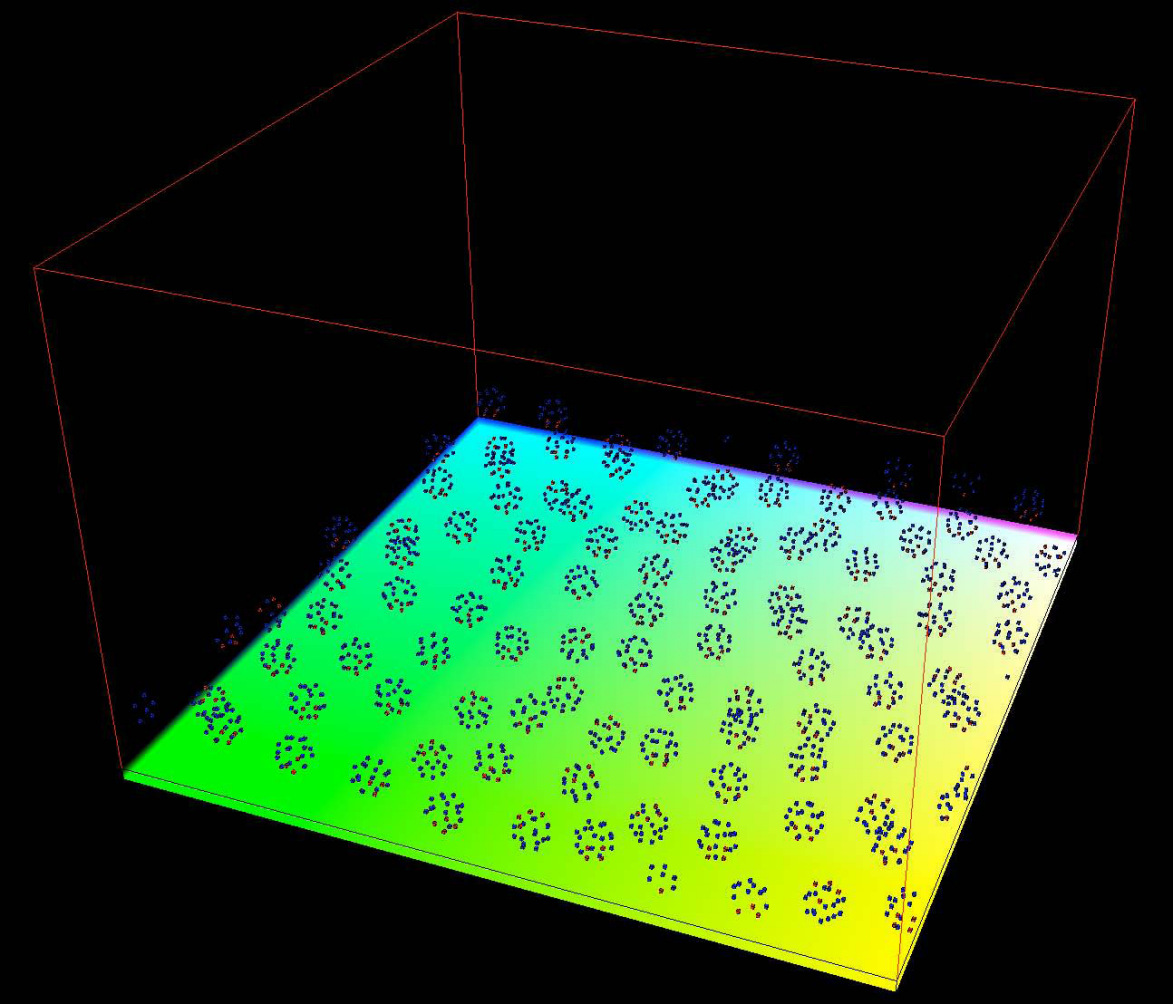
**Single layer of epidermal cells**. 3D visualization of 100 cells each with 20 subcellular elements and periodic boundary conditions forming a single layer of the epidermis. The greenish-yellow plane under the cells represents the basement membrane. Red subcellular elements have an adhesive force term with the basement membrane, and blue subcellular elements indicate the basal cellular state.

**Figure 4:**
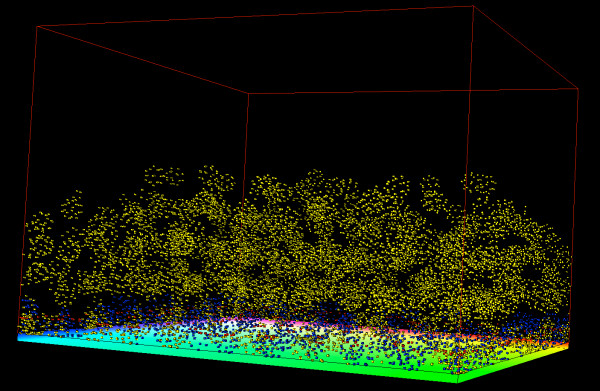
**Multiple layers of epidermal cells**. 3D visualization of full model after running for 130000 iterations showing 535 cells forming multiple layers of the epidermis. The model started with 100 cells in a single layer as shown in Figure 3 and proceeded through about three cell divisions. The greenish-yellow plane under the cells represents the basement membrane. Red subcellular elements have an adhesive force term with the basement membrane, while blue subcellular elements indicate the basal cellular state and yellow subcellular elements indicate the suprabasal cellular state.

Figures 3 and 4 show 3D visualization results from running simulations of the full model with initial cell population of 100 cells and periodic boundary conditions. Figure 3 is a starting condition where the cells form a single layer adhering to the basement membrane. Figure 4 is at the end of the simulation where the cells have gone through a few rounds of division forming multiple layers of the epidermis. Subcellular elements are colored red if they are of the type for basement membrane adhesion. Otherwise, elements are colored blue if they are in the basal cellular state or colored yellow if they are in the suprabasal cellular state.

## Conclusions

The purpose of this article has been to explore the feasibility of using the technology capabilities of GPUs to model complex, integrative multicellular biological phenomena. As a case study, we constructed a 3D model of epidermal development incorporating cell shape, movement and adhesion using the subcellular element method, cell growth and division, and an intracellular gene network within each cell coupled to cell-cell and cell-environment interactions. We implemented the epidermal model in GPU algorithms, and in the process we discussed various programmatic issues and provided a set of design guidelines that we hope will be instructive to other modelers, both to avoid common pitfalls as well as to exploit the GPU for performance gain.

What we have not explored are the issues of multiscale modeling. For our epidermal model, we have simulated the cell shape and movement actions on the same time scale as the intracellular gene network. In reality, it is more likely to assume that the cell shape and movement actions operate on a much longer time scale than the gene network, thus allowing a time scale separation. This might be implemented, as is commonly done in agent-based models [38], through nested loops where the gene network operates in the innermost fast loop while the cell actions operate in the outermost slow loop. This should result in some significant savings in execution time as the subcellular element method dominates the computation in our epidermal model.

While we have focused on the subcellular element method for the representation of cells, the Delaunay-Object-Dynamics method [45] can also potentially take great advantage of GPUs due to its use of Voronoi cells. Voronoi tessellation is a well-studied topic in computational geometry with many GPU algorithms [91,92], so use of these algorithms might allow an even greater number of cells to be modeled. What needs more investigation is how well stochastic modeling methods can utilize GPU hardware. Markov chain based methods such as the cellular Potts model alter the probability distribution of the whole system when a state change occur, so individual cells cannot act in parallel without taking the statistical correlations into account.

Despite the obvious speed advantages of GPUs for data-parallel programming, they have some definite limitations compared to other parallel architectures. Most notably is that GPUs have essentially no synchronization or communication capability between concurrent threads. Algorithms where threads communicate intermediate results to other threads cannot be directly used. Though some GPUs do offer a simplistic block-level synchronization, it is restricted to a subset of threads (based upon grid/block decomposition) so algorithms have to be specially designed with that constraint, and even then it is a code barrier synchronization and has no communication capabilities. Another limitation is the lack of semaphore or locking primitives for shared writable memory, so threads have no mechanism to coordinate memory writes and prevent one thread from overwriting another. More recent GPUs provide atomic write functions, while this does not provide the full capability of locking, it does allow lock-free data structures to be considered. GPUs also have strict memory limitations. The amount of memory available on GPUs is much less than for CPUs; the Nvidia GTX 285 has 1 GB which is plenty sufficient for our model, but GPUs lack virtual memory capability so the available memory is a hard upper limit and has to be used wisely.

We have demonstrated that GPU algorithms can be used to great advantage to speedup the execution time of integrative multicellular biological models. There are further improvements that can be made to our GPU code. For example, we only use constant and global memory, so using shared memory can greatly speedup memory access. This would have the most benefit in the kernels for the subcellular element method as they are the most computationally expensive, however the code can become more complicated and requires careful design to use effectively. Also some of our kernels do not maximize GPU occupancy as well as they could, so we can further fine-tune these kernels to either use more concurrent threads or reduce the number of registers being used. We hope in the future to continue developing our epidermal model and use it to investigate putative underlying mechanisms responsible for the processes of stratification and homeostasis as well as the role that environmental and cellular interactions play in stem cell maintenance and proliferation.

## Authors' contributions

SC conceived the study, participated in its design, performed the experiments and helped draft the manuscript. BL and XD participated in the design of the study. QN participated in the design of the study and helped draft the manuscript. All authors read and approved the final manuscript.

## Appendix

The following algorithms are provided in sufficient detail so the various parameters and GPU-related functions calls can be examined, however many details such as variable declarations, parameter definitions, error checking, utility functions, etc. have been eliminated for brevity. The full source code is provided as Additional file 1. Also as we go from one algorithm to the next, adding new functionality along the way, we only show the new code while leaving just comments for the previous code.

### Algorithm 1

void SEM(int numOfCells, int maxCells, int *elements, int maxElements,

      float *hostX, float *hostY, float *hostZ, float *hostType,

      float *hostParameters, float dt, float timeSteps)

{

   //Allocate device memory

   //cells and elements

   cudaMalloc(&numOfElements, maxCells * sizeof(int));

   cudaMallocPitch(&elementType, &pitch, maxCells * sizeof(int), maxElements);

   cudaMallocPitch(&X, &pitch, maxCells * sizeof(float), maxElements);

   cudaMallocPitch(&X_F1, &pitch, maxCells * sizeof(float), maxElements);

   cudaMallocPitch(&X_F2, &pitch, maxCells * sizeof(float), maxElements);

   cudaMallocPitch(&Y, &pitch, maxCells * sizeof(float), maxElements);

   cudaMallocPitch(&Y_F1, &pitch, maxCells * sizeof(float), maxElements);

   cudaMallocPitch(&Y_F2, &pitch, maxCells * sizeof(float), maxElements);

   cudaMallocPitch(&Z, &pitch, maxCells * sizeof(float), maxElements);

   cudaMallocPitch(&Z_F1, &pitch, maxCells * sizeof(float), maxElements);

   cudaMallocPitch(&Z_F2, &pitch, maxCells * sizeof(float), maxElements);

   //Copy host memory to device memory

   //spatial positions

   cudaMemcpy(numOfElements, elements, maxCells * sizeof(int),

         cudaMemcpyHostToDevice);

   cudaMemcpy2D(elementType, pitch, hostType, maxCells * sizeof(int),

         maxCells * sizeof(int), maxElements, cudaMemcpyHostToDevice);

   cudaMemcpy2D(X, pitch, hostX, maxCells * sizeof(float),

         maxCells * sizeof(float), maxElements, cudaMemcpyHostToDevice);

   cudaMemcpy2D(Y, pitch, hostY, maxCells * sizeof(float),

         maxCells * sizeof(float), maxElements, cudaMemcpyHostToDevice);

   cudaMemcpy2D(Z, pitch, hostZ, maxCells * sizeof(float),

         maxCells * sizeof(float), maxElements, cudaMemcpyHostToDevice);

   //parameters

   cudaMemcpyToSymbol(skin_parameters, hostParameters, 100 * sizeof(float), 0,

            cudaMemcpyHostToDevice);

   //execute kernel

   for (t = 0; t < timeSteps; ++t) {

      //movement kernels

      sem_kernel_F1 < < < blocksPerGrid, threadsPerBlock > > > (numOfElements, X, X_F1,

         X_F2, Y, Y_F1, Y_F2, Z, Z_F1, Z_F2, elementType,

         pitch/sizeof(float), numOfCells, maxCells, maxElements, dt);

      sem_kernel_F2 < < < blocksPerGrid, threadsPerBlock > > > (numOfElements, X, X_F1,

         X_F2, Y, Y_F1, Y_F2, Z, Z_F1, Z_F2, elementType,

         pitch/sizeof(float), numOfCells, maxCells, maxElements, dt);

      cudaMemcpy2D(X, pitch, X_F2, pitch, maxCells * sizeof(float),

         maxElements, cudaMemcpyDeviceToDevice);

      cudaMemcpy2D(Y, pitch, Y_F2, pitch, maxCells * sizeof(float),

         maxElements, cudaMemcpyDeviceToDevice);

      cudaMemcpy2D(Z, pitch, Z_F2, pitch, maxCells * sizeof(float),

         maxElements, cudaMemcpyDeviceToDevice);

}

   //Copy result to host memory

   cudaMemcpy2D(hostX, maxCells * sizeof(float), X, pitch,

         maxCells * sizeof(float), maxElements, cudaMemcpyDeviceToHost);

   cudaMemcpy2D(hostY, maxCells * sizeof(float), Y, pitch,

         maxCells * sizeof(float), maxElements, cudaMemcpyDeviceToHost);

   cudaMemcpy2D(hostZ, maxCells * sizeof(float), Z, pitch,

         maxCells * sizeof(float), maxElements, cudaMemcpyDeviceToHost);

}

   __global__ void

   sem_kernel_F1(int *numOfElements, float *X, float *X_F1, float *X_F2, float *Y,

         float *Y_F1, float *Y_F2, float *Z, float *Z_F1, float *Z_F2,

         int *elementType, size_t pitch, int numOfCells, int maxCells,

         int maxElements, float dt)

{

   int cellNum = blockIdx.x * blockDim.x + threadIdx.x;

   int elemNum = blockIdx.y * blockDim.y + threadIdx.y;

   if (cellNum > = numOfCells) return;

   if (elemNum > = numOfElements[cellNum]) return;

   //intracellular

   float intraX = 0.0;

   float intraY = 0.0;

   float intraZ = 0.0;

   for (k = 0; k < numOfElements[cellNum]; ++k) {

      if (k == elemNum) continue;

      r = dist(X[elemNum*pitch+cellNum], Y[elemNum*pitch+cellNum],

         Z[elemNum*pitch+cellNum], X[k*pitch+cellNum],

         Y[k*pitch+cellNum], Z[k*pitch+cellNum]);

      V = MORSE(r, INTRA_U0, INTRA_ETA0, INTRA_U1, INTRA_ETA1);

      intraX += V * (X[elemNum*pitch+cellNum] - X[k*pitch+cellNum]);

      intraY += V * (Y[elemNum*pitch+cellNum] - Y[k*pitch+cellNum]);

      intraZ += V * (Z[elemNum*pitch+cellNum] - Z[k*pitch+cellNum]);

   #if PERIODIC_BOUNDARY

      intracellular_mirror(elemNum, cellNum, pitch, k, &intraX, &intraY,

            &intraZ, X, Y, Z);

   #endif

   }

   //intercellular

   float interX = 0.0;

   float interY = 0.0;

   float interZ = 0.0;

   for (j = 0; j < numOfCells; ++j) {

      if (j == cellNum) continue;

      for (k = 0; k < numOfElements[j]; ++k) {

       r = dist(X[elemNum*pitch+cellNum], Y[elemNum*pitch+cellNum],

         Z[elemNum*pitch+cellNum],

         X[k*pitch+j], Y[k*pitch+j], Z[k*pitch+j]);

      if (r <= INTER_DIST) {

       V = P_MORSE(r, INTER_U0, INTER_ETA0, INTER_U1, INTER_ETA1);

       interX += V * (X[elemNum*pitch+cellNum] - X[k*pitch+j]);

       interY += V * (Y[elemNum*pitch+cellNum] - Y[k*pitch+j]);

       interZ += V * (Z[elemNum*pitch+cellNum] - Z[k*pitch+j]);

      }

   #if PERIODIC_BOUNDARY

      intercellular_mirror(elemNum, cellNum, pitch, k, j,

            &interX, &interY, &interZ, X, Y, Z);

   #endif

      }

   }

   //basement membrane

   if (elementType[elemNum*pitch+cellNum] == 1) {

      r = dist(X[elemNum*pitch+cellNum], Y[elemNum*pitch+cellNum],

         Z[elemNum*pitch+cellNum], X[elemNum*pitch+cellNum],

         Y[elemNum*pitch+cellNum], 0);

   V = MORSE(r, INTRA_U0, INTRA_ETA0, INTRA_U1, INTRA_ETA1);

   interZ += V * (Z[elemNum*pitch+cellNum] - 0);

}

   //update

   X_F1[elemNum*pitch+cellNum] = X[elemNum*pitch+cellNum]

            + 0.5 * dt * (intraX + interX);

   Y_F1[elemNum*pitch+cellNum] = Y[elemNum*pitch+cellNum]

            + 0.5 * dt * (intraY + interY);

   Z_F1[elemNum*pitch+cellNum] = Z[elemNum*pitch+cellNum]

            + 0.5 * dt * (intraZ + interZ);

}

### Algorithm 2

void SEM(int numOfCells, int maxCells, int *elements, int maxElements,

      float *hostX, float *hostY, float *hostZ, float *hostType,

      float *hostParameters, float dt, float timeSteps)

{

   //Allocate device memory

   //cells and elements ..

   //cell centers

   cudaMalloc(&cellCenterX, maxCells * sizeof(float));

   cudaMalloc(&cellCenterY, maxCells * sizeof(float));

   cudaMalloc(&cellCenterZ, maxCells * sizeof(float));

   //Copy host memory to device memory

   //spatial positions ..

   //parameters ..

   //execute kernel

   for (t = 0; t < timeSteps; ++t) {

     //movement kernels

     skin_center_kernel < < < blocksPerGrid1 D,threadsPerBlock1D > > > (numOfElements,

            X, Y, Z, elementType, cellCenterX, cellCenterY,

            cellCenterZ, pitch/sizeof(float), numOfCells,

            maxCells, maxElements);

     sem_kernel_F1 < < < blocksPerGrid, threadsPerBlock > > > (numOfElements, X, X_F1,

         X_F2, Y, Y_F1, Y_F2, Z, Z_F1, Z_F2, elementType,

         pitch/sizeof(float), numOfCells, maxCells, maxElements, dt);

     sem_kernel_F2 < < < blocksPerGrid, threadsPerBlock > > > (numOfElements, X, X_F1,

         X_F2, Y, Y_F1, Y_F2, Z, Z_F1, Z_F2, elementType,

         pitch/sizeof(float), numOfCells, maxCells, maxElements, dt);

   }

     //Copy result to host memory ..

}

   __global__ void

   skin_center_kernel(int *numOfElements, float *X, float *Y, float *Z,

            int *elementType, float *cellCenterX, float *cellCenterY,

            float *cellCenterZ, size_t pitch,

            int numOfCells, int maxCells, int maxElements, float dt)

{

     int cellNum = blockIdx.x * blockDim.x + threadIdx.x;

     int elemNum;

     if (cellNum > = numOfCells) return;

     float cX = 0.0;

     float cY = 0.0;

     float cZ = 0.0;

     float minX, maxX;

     float minY, maxY;

     minX = X[cellNum];

     maxX = X[cellNum];

     minY = Y[cellNum];

     maxY = Y[cellNum];

     for (elemNum = 0; elemNum < numOfElements[cellNum]; ++elemNum) {

      cX += X[elemNum*pitch+cellNum];

      cY += Y[elemNum*pitch+cellNum];

      cZ += Z[elemNum*pitch+cellNum];

      if (X[elemNum*pitch+cellNum] < minX) minX = X[elemNum*pitch+cellNum];

      if (X[elemNum*pitch+cellNum] > maxX) maxX = X[elemNum*pitch+cellNum];

      if (Y[elemNum*pitch+cellNum] < minY) minY = Y[elemNum*pitch+cellNum];

      if (Y[elemNum*pitch+cellNum] > maxY) maxY = Y[elemNum*pitch+cellNum];

}

   cX = cX/(float)numOfElements[cellNum];

   cY = cY/(float)numOfElements[cellNum];

   cZ = cZ/(float)numOfElements[cellNum];

   //handle special case when cell is split across periodic boundary

   if ((maxX - minX) > (BOUNDARY_X/2)) {

      cX = 0;

      for (elemNum = 0; elemNum < numOfElements[cellNum]; ++elemNum) {

       if (X[elemNum*pitch+cellNum] > (BOUNDARY_X/2))

         cX += X[elemNum*pitch+cellNum] - BOUNDARY_X;

       else

         cX += X[elemNum*pitch+cellNum];

}

      cX = cX/(float)numOfElements[cellNum];

      if (cX < 0) cX += BOUNDARY_X;

}

   if ((maxY - minY) > (BOUNDARY_Y/2)) {

      cY = 0;

      for (elemNum = 0; elemNum < numOfElements[cellNum]; ++elemNum) {

       if (Y[elemNum*pitch+cellNum] > (BOUNDARY_Y/2))

         cY += Y[elemNum*pitch+cellNum] - BOUNDARY_Y;

       else

         cY += Y[elemNum*pitch+cellNum];

}

      cY = cY/(float)numOfElements[cellNum];

      if (cY < 0) cY += BOUNDARY_Y;

}

   cellCenterX[cellNum] = cX;

   cellCenterY[cellNum] = cY;

   cellCenterZ[cellNum] = cZ;

}

   __global__ void

   sem_kernel_F1(int *numOfElements, float *X, float *X_F1, float *X_F2, float *Y,

            float *Y_F1, float *Y_F2, float *Z, float *Z_F1, float *Z_F2,

            float *cX, float *cY, float *cZ,

            int *elementType, size_t pitch, int numOfCells, int maxCells,

            int maxElements, float dt)

   {

   int cellNum = blockIdx.x * blockDim.x + threadIdx.x;

   int elemNum = blockIdx.y * blockDim.y + threadIdx.y;

   if (cellNum > = numOfCells) return;

   if (elemNum > = numOfElements[cellNum]) return;

   //intracellular ..

   //intercellular

   float interX = 0.0;

   float interY = 0.0;

   float interZ = 0.0;

   for (j = 0; j < numOfCells; ++j) {

      if (j == cellNum) continue;

      //check if cell centers are close enough

      r = dist(cX[cellNum], cY[cellNum], cZ[cellNum], cX[j], cY[j], cZ[j]);

      if (r > INTER_DIST) continue;

      for (k = 0; k < numOfElements[j]; ++k) {

       r = dist(X[elemNum*pitch+cellNum], Y[elemNum*pitch+cellNum],

         Z[elemNum*pitch+cellNum],

         X[k*pitch+j], Y[k*pitch+j], Z[k*pitch+j]);

       if (r <= INTER_DIST) {

         V = P_MORSE(r, INTER_U0, INTER_ETA0, INTER_U1, INTER_ETA1);

         interX += V * (X[elemNum*pitch+cellNum] - X[k*pitch+j]);

         interY += V * (Y[elemNum*pitch+cellNum] - Y[k*pitch+j]);

         interZ += V * (Z[elemNum*pitch+cellNum] - Z[k*pitch+j]);

}

   #if PERIODIC_BOUNDARY

      intercellular_mirror(elemNum, cellNum, pitch, k, j,

                  &interX, &interY, &interZ, X, Y, Z);

   #endif

   }

}

   //basement membrane ..

   //update ..

}

### Algorithm 3

void SEM(int numOfCells, int maxCells, int *elements, int maxElements,

      float *hostX, float *hostY, float *hostZ, float *hostType,

      float *hostParameters, int numOfSpecies, float *speciesData,

      float dt, float timeSteps)

{

   //Allocate device memory

   //cells and elements ..

   //cell centers ..

   //intracellular gene network

   cudaMalloc(&neighborNum, maxCells * sizeof(float));

   cudaMallocPitch(&sData, &pitch, maxCells * sizeof(float), numOfSpecies);

   cudaMallocPitch(&sData_F1, &pitch, maxCells * sizeof(float), numOfSpecies);

   cudaMallocPitch(&sData_F2, &pitch, maxCells * sizeof(float), numOfSpecies);

   cudaMallocPitch(&neighborData, &pitch, maxCells * sizeof(float),

            numOfSpecies);

   //Copy host memory to device memory ..

   //spatial positions ..

   //parameters ..

   //intracellular gene network

   cudaMemcpy2D(sData, pitch, speciesData, maxCells * sizeof(float),

         maxCells * sizeof(float), numOfSpecies, cudaMemcpyHostToDevice);

   //execute kernel

   for (t = 0; t < timeSteps; ++t) {

      //movement kernels ..

      //gene network kernels

      skin_neighbor_kernel < < < blocksPerGrid1 D,threadsPerBlock1D > > > (numOfElements,

            cellCenterX, cellCenterY, cellCenterZ, elementType,

            neighborNum, pitch/sizeof(float),

            sData, sData_F1, sData_F2, neighborData, numOfCells,

            maxCells, maxElements);

      skin_kernel_F1 < < < blocksPerGrid1 D,threadsPerBlock1D > > > (numOfElements,

         X, Y, Z, elementType, neighborNum, pitch/sizeof(float),

         sData, sData_F1, sData_F2, neighborData,

         numOfCells, maxCells, maxElements, dt);

      skin_kernel_F2 < < < blocksPerGrid1 D,threadsPerBlock1D > > > (numOfElements,

         X, Y, Z, elementType, neighborNum, pitch/sizeof(float),

         sData, sData_F1, sData_F2, neighborData,

         numOfCells, maxCells, maxElements, dt);

   }

   //Copy result to host memory ..

}

   __global__ void

   skin_neighbor_kernel(int *numOfElements, float *cellCenterX,

            float *cellCenterY, float *cellCenterZ, int *elementType,

            int *neighborNum, size_t pitch,

            float *speciesData, float *speciesData_F1,

            float *speciesData_F2, float *neighborData,

            int numOfCells, int maxCells, int maxElements)

{

   int cellNum = blockIdx.x * blockDim.x + threadIdx.x;

   int j;

   if (cellNum > = numOfCells) return;

   //totals from neighbors

   float neighbor_NOTCH = 0.0;

   float neighbor_DELTA = 0.0;

   float neighbor_BOUND = 0.0;

   int numOfNeighbors = 0;

   for (j = 0; j < numOfCells; ++j) {

      if (j == cellNum) continue;

      if (distance_check(cellCenterX[cellNum], cellCenterY[cellNum],

               cellCenterZ[cellNum], cellCenterX[j],

               cellCenterY[j], cellCenterZ[j],

               S_XY_TOT, NEIGHBOR_DIST) != S_NONE) {

         ++numOfNeighbors;

         neighbor_NOTCH += speciesData[NOTCH_species*pitch+j];

         neighbor_DELTA += speciesData[DELTA_species*pitch+j];

         neighbor_BOUND += speciesData[BOUND_species*pitch+j];

   }

}

   neighborNum[cellNum] = numOfNeighbors;

   neighborData[NOTCH_species*pitch+cellNum] = neighbor_NOTCH;

   neighborData[DELTA_species*pitch+cellNum] = neighbor_DELTA;

   neighborData[BOUND_species*pitch+cellNum] = neighbor_BOUND;

}

   __global__ void

   skin_kernel_F1(int *numOfElements, float *X, float *Y, float *Z,

            int *elementType, int *neighborNum, size_t pitch,

            float *speciesData, float *speciesData_F1,

            float *speciesData_F2, float *neighborData,

            int numOfCells, int maxCells, int maxElements, float dt)

{

   int cellNum = blockIdx.x * blockDim.x + threadIdx.x;

   if (cellNum > = numOfCells) return;

   float interactionValue, F1_val;

   //calculate F1

   float NOTCH_val = speciesData[NOTCH_species*pitch+cellNum];

   float DELTA_val = speciesData[DELTA_species*pitch+cellNum];

   float BOUND_val = speciesData[BOUND_species*pitch+cellNum];

   float BMA_val = speciesData[BMA_species*pitch+cellNum];

   float OVOL1_val = speciesData[OVOL1_species*pitch+cellNum];

   float OVOL2_val = speciesData[OVOL2_species*pitch+cellNum];

   float CMYC_val = speciesData[CMYC_species*pitch+cellNum];

   //averages from neighbors

   float neighbor_NOTCH = 0.0;

   float neighbor_DELTA = 0.0;

   float neighbor_BOUND = 0.0;

   int numOfNeighbors = 0;

   neighbor_NOTCH = neighborData[NOTCH_species*pitch+cellNum];

   neighbor_DELTA = neighborData[DELTA_species*pitch+cellNum];

   neighbor_BOUND = neighborData[BOUND_species*pitch+cellNum];

   numOfNeighbors = neighborNum[cellNum];

   if (numOfNeighbors != 0) {

      neighbor_NOTCH = neighbor_NOTCH/(float)numOfNeighbors;

      neighbor_DELTA = neighbor_DELTA/(float)numOfNeighbors;

      neighbor_BOUND = neighbor_BOUND/(float)numOfNeighbors;

}

   //NOTCH

   interactionValue = HILL(BOUND_val, NOTCH_pmin_BOUND, NOTCH_pmax_BOUND,

               NOTCH_c_BOUND, NOTCH_h_BOUND);

   interactionValue *= HILL(OVOL2_val, NOTCH_pmin_OVOL2, NOTCH_pmax_OVOL2,

               NOTCH_c_OVOL2, NOTCH_h_OVOL2);

   F1_val = NOTCH_val + 0.5 * dt * ((-KA) * NOTCH_val * neighbor_DELTA

               + KD * BOUND_val - DF * NOTCH_val

               + interactionValue);

   if (F1_val < 0.0) F1_val = 0;

   speciesData_F1[NOTCH_species*pitch+cellNum] = F1_val;

   //DELTA

   interactionValue = HILL(NOTCH_val, DELTA_pmin_BOUND, DELTA_pmax_BOUND,

            DELTA_c_BOUND, DELTA_h_BOUND);

   F1_val = DELTA_val + 0.5 * dt * ((-KA) * DELTA_val * neighbor_NOTCH

               + KD * neighbor_BOUND - DA * DELTA_val

               + interactionValue);

   if (F1_val < 0.0) F1_val = 0;

   speciesData_F1[DELTA_species*pitch+cellNum] = F1_val;

   //BOUND RECEPTOR

   F1_val = BOUND_val + 0.5 * dt * (KA * NOTCH_val * neighbor_DELTA

               - KD * BOUND_val - KI * BOUND_val);

   if (F1_val < 0.0) F1_val = 0;

   speciesData_F1[BOUND_species*pitch+cellNum] = F1_val;

   //Basement Membrane Adhesion

   speciesData_F1[BMA_species*pitch+cellNum] =

         speciesData[BMA_species*pitch+cellNum];

   //OVOL1

   interactionValue = HILL(OVOL2_val, OVOL1_pmin_OVOL2, OVOL1_pmax_OVOL2,

            OVOL1_c_OVOL2, OVOL1_h_OVOL2);

   interactionValue *= HILL(BMA_val, OVOL1_pmin_BMA, OVOL1_pmax_BMA,

            OVOL1_c_BMA, OVOL1_h_BMA);

   F1_val = OVOL1_val + 0.5 * dt * (interactionValue - OVOL1_decay * OVOL1_val);

   if (F1_val < 0.0) F1_val = 0;

   speciesData_F1[OVOL1_species*pitch+cellNum] = F1_val;

   //OVOL2

   interactionValue = HILL(OVOL1_val, OVOL2_pmin_OVOL1, OVOL2_pmax_OVOL1,

            OVOL2_c_OVOL1, OVOL2_h_OVOL1);

   interactionValue *= HILL(0.4, 0.0, 1.0, 1.0, 1.0);//TGF-beta

   F1_val = OVOL2_val + 0.5 * dt * (interactionValue - OVOL2_decay * OVOL2_val);

   if (F1_val < 0.0) F1_val = 0;

   speciesData_F1[OVOL2_species*pitch+cellNum] = F1_val;

   //CMYC

   interactionValue = HILL(OVOL2_val, CMYC_pmin_OVOL2, CMYC_pmax_OVOL2,

            CMYC_c_OVOL2, CMYC_h_OVOL2);

   F1_val = CMYC_val + 0.5 * dt * (interactionValue - CMYC_decay * CMYC_val);

   if (F1_val < 0.0) F1_val = 0;

   speciesData_F1[CMYC_species*pitch+cellNum] = F1_val;

}

## Supplementary Material

Additional file 1 **Source code for 3D epidermal model using GPU algorithms**. CUDA source code is provided in three separate directories for the three different models (movement, network, full) presented in the paper. The Readme.txt file provides additional information. Click here for file
